# VetCompanion: response and clarifications

**DOI:** 10.5195/jmla.2017.187

**Published:** 2017-07-01

**Authors:** Matthew Paul Mauer, Jennifer Garcia

**To the editor,** we are writing in response to the recent VetCompanion review [1]. The positive suggestions made by the author are appreciated. We agree the formulary provides substantial value. We plan to improve the search functionality by incorporating Boolean operators and exclusively utilizing tags applied to each topic, allowing more refined searches based on multiple symptoms. Our content is continuously growing, and the two surgical topics identified were added last fall. The VetMed Resource is now used in developing and updating topics. We also have an increasing number of specialists who serve as peer reviewers but were not listed on the website, which will be revised to prevent possible confusion. We also note that VetCompanion is mobile optimized: an icon uploaded to mobile devices allows it to function like a native app.

However, we have concerns about several points raised by the author of the review, who felt the content is not detailed enough to be clinically useful. In a recent user survey, over 90% of respondents stated that they would recommend VetCompanion to colleagues, which suggests they do not share this view.

Of greater concern are two points regarding “omissions and unusual recommendations.” Recommendations are based on current peer-reviewed sources in the veterinary literature. For example, follow-up thoracic radiography is recommended for congestive heart failure as it is the optimal modality to assess pulmonary edema [2]. We acknowledge that some users might appreciate details regarding the use of echocardiography to evaluate underlying cardiac disease, although recommendations regarding specialist referral are included in other sections. In addition, regarding pancreatitis, the author states “methadone is rarely used in small animal practice in the United States.” When choosing the best analgesic for these patients, opioids such as methadone are often recommended for pain control [3, 4]. Methadone is actually used frequently for pain management in many private practices and university settings, as it causes less sedation and is less likely to cause vomiting than other opioids [5].

The author then suggests, we feel unfairly, that the value of VetCompanion is diminished because methodology information is not available directly on the user page and links are not provided for all evidence. We believe users can easily reference methodology information where it is transparently presented on the public website. We made a decision to exclude links to lower-level studies, although we may eventually add such links as a courtesy.

Another concern is the comparison with Vetstream [6], which offers no discussion of methodology. Vetstream provides no explanation of any evidence-based methodology for developing content and includes no rigorous evidence ratings like VetCompanion does. They also provide no list of their reviewers. Of greatest concern, Vetstream accepts advertising, which presents significant potential for biases. VetCompanion, similar to human medicine resources, accepts no advertising. This is critical for any evidence-based point-of-care resource. Thus, the omission of these issues calls the accuracy of the author’s comparison into question.

VetCompanion is indeed a “work in progress” and always will be. The very nature of such technologies is constant change. Thus, the iteration of VetCompanion that was reviewed is not the iteration being used today; each week, a new iteration evolves. We are adding new topics and updating content on an ongoing basis, constantly improving the user experience with the goal of providing the most innovative, highest quality, unbiased, evidence-based point-of-care content available for veterinary clinicians.
